# DTL-YOLO: a lightweight model for real-time tobacco leaf damage detection and mechanical harvesting quality assessment

**DOI:** 10.3389/fpls.2026.1971289

**Published:** 2026-10-16

**Authors:** Yue Luo, Jun Yang, Chenhui Zhu, Feng Liu, Wanzhang Wang, Dan Wei

**Affiliations:** 1 College of Mechanical and Electrical Engineering, Henan Agricultural University, Zhengzhou, China; 2 State Key Laboratory of Intelligent Agricultural Power Equipment, Luoyang, China

**Keywords:** damaged tobacco leaf detection, layered tobacco harvester, lightweight networks, quality assessment, YOLOv8

## Abstract

Tobacco is an important industrial crop worldwide. Leaf damage caused by mechanical harvesting severely reduces tobacco leaf quality and commercial value, hindering the promotion of mechanized tobacco production. Real-time and accurate detection of mechanical damage in fresh tobacco leaves is essential for harvesting quality assessment. Nevertheless, current industrial evaluation relies largely on post-harvest manual sampling and statistics, which suffers from high labor costs, low efficiency and strong subjective bias. Automated and real-time approaches for damage detection during tobacco harvesting remain insufficiently investigated. To address this challenge, this study proposes a novel lightweight tobacco leaf damage detection model, termed DTL-YOLO (Damaged Tobacco Leaf-YOLO), based on an improved YOLOv8 architecture. Fresh tobacco leaves harvested by a layered tobacco harvester were used as the research object to enable damage detection under realistic harvesting conditions. The proposed model adopts StarNet as a lightweight backbone and integrates an improved Bidirectional Feature Pyramid Network (BiFPN) for feature fusion, while an adaptive downsampling module (ADown) is incorporated to enhance boundary perception of damaged leaf regions. With only 5.4 GFLOPs and 1.17M parameters, the proposed method attains 80.9% precision, 81.0% recall, and mean average precision of 88.4% in the evaluation. Compared with the YOLOv8n baseline, the proposed model improves recall by 1.9 percentage points and mAP_50_ by 1.8 percentage points while effectively reducing computational complexity and model size. This approach enables real-time detection of damaged fresh tobacco leaves under mechanical harvesting conditions, thereby facilitating harvest-quality assessment and supporting the design of low-damage layered tobacco harvesters.

## 1 Introduction

Tobacco (*Nicotiana tabacum* L.) is an economically important crop widely cultivated worldwide (Murtaza et al., 2025; Xing et al., 2025). With the development of smart agriculture, intelligent perception and non-destructive detection technologies have become increasingly important for rapid assessment of crop quality and operational processes. Traditional tobacco leaf harvesting relies heavily on manual labor, resulting in heavy labor intensity, low harvesting efficiency and elevated production costs. Advances in science and technology have driven the development and wider adoption of semi-automatic and fully automatic tobacco harvesting systems, gradually replacing traditional manual harvesting methods (Sun et al., 2024; Tian et al., 2023). Among them, the fully automatic tobacco layered harvester has become the mainstream option in the current tobacco harvesting industry due to its advantages of high efficiency and precision. During harvesting, the tobacco leaf damage rate provides a practical measure of the operational performance of layered harvesting machinery (Yu et al., 2019). Mechanical damage may reduce harvested leaf quality by causing tissue loss, breakage, and deformation, thereby affecting subsequent handling and curing processes. Therefore, timely and accurate detection of damaged leaves is essential for quantitative harvesting quality assessment and provides important information for evaluating harvesting performance.

Recent advances in deep learning have broadened its application in agricultural visual perception, encompassing leaf object detection, plant disease identification, and phenotypic analysis (Luo et al., 2025; Poblete-Echeverría et al., 2025; Shehu et al., 2025), thereby supporting the assessment of fresh tobacco leaf quality. In the field of leaf object detection, Zhang et al. (2021) focused on sweet potatoes as the research object and achieved accurate recognition of leaf shapes in field environments through detection of the main leaf skeleton and estimation of leaf orientation. However, this study did not include leaves with invisible main veins or damaged leaves in the detection scope. Zhou et al. (2025) completed the detection and counting of rice leaves in field environments by improving the YOLOv5s algorithm. Similarly, Li et al. (2023b) and Wang et al. (2025) achieved the detection of leaf numbers in wheat seedlings and the leaf counting of crops such as maize seedlings, respectively. In the field of plant disease detection, practical achievements have been made in detecting leaf diseases of protected vegetables and crops grown in natural environments (Li et al., 2022; Tang et al., 2023). Among them, Li et al. (2022) introduced a detection model for crop pests and diseases using YOLOv5s, which can identify common crop diseases in tomato leaves, cucumber leaves, and Chinese cabbage leaves. Tang et al. (2023) constructed PLPNet, a tomato leaf disease detection model based on the lightweight YOLOX-S, which improves detection accuracy while ensuring detection speed, thus meeting the real-time requirements for tomato leaf disease detection. Zenkl et al. (2025) utilized deep learning techniques to complete field detection and quantification of wheat foliar diseases. Jiang et al. (2021) applied multi-task transfer learning to this field, enabling the concurrent recognition of leaf diseases in both rice and wheat crops. In the field of leaf phenotypic analysis, Guan et al. (2024) proposed the DBi-YOLOv8 algorithm for detecting canopy organs of maize plants and developed the LTNS algorithm to count leaves and ears on the plants. Chen et al. (2025) combined high-resolution UAV images with YOLOv8n for rice seedling identification and conducted large-scale counting of rice seedlings.

To achieve real-time and non-destructive detection under field conditions, lightweight deep learning models have received increasing attention in agricultural visual applications because they provide a balance between detection performance and computational efficiency. By optimizing model architectures and improving computational efficiency, such approaches enable reliable visual analysis while reducing resource requirements, thereby facilitating real-time deployment in agricultural production environments. For lotus leaf pest and disease detection, Liu et al. (2024) presented a lightweight YOLOv8 based model that improved mAP by 1.6% and reduced parameters by 0.2M, supporting the effectiveness of lightweight design for detection. In the field of tobacco leaf detection, Chen et al. (2023b) developed a low-cost tobacco leaf maturity identification system, which assists in harvesting decisions based on machine vision and deep learning. Wang et al. (2023b) realized the judgment of tobacco leaf maturity grades in field environments based on an improved YOLOv5 framework, with the recognition accuracy of all parts exceeding 90%. Wu et al. (2024) developed TobaccoNet, which considers fine-grained information and effectively fuses its features, achieving a recognition accuracy of 96.67% for fresh tobacco leaf maturity. Dai et al. (2024) designed a multi-scale branch attention network (MSBANet) for tobacco leaf maturity identification. Its lightweight version, MSBANet-S, achieves an accuracy of 88.2% with only 1.7 GFLOPs, while MSBANet-L achieves 89.6% accuracy with 7.1 GFLOPs, providing a solution for real-time field detection. The aforementioned studies have facilitated the application of lightweight models in complex agricultural scenarios. However, existing studies mainly focus on visual characteristics associated with tobacco maturity and diseases, whereas mechanical harvesting damage presents distinct patterns such as irregular breakage, missing regions, and local deformation. Therefore, dedicated detection methods are required for accurate identification of mechanically damaged tobacco leaves under harvesting conditions. Studies on other agricultural products have demonstrated the importance of mechanical damage detection and non-destructive evaluation for quality assessment (Castillo-Girones et al., 2024; Feng et al., 2023; Wang B. et al., 2024).

Despite substantial advances in deep learning methods for plant leaf detection, studies that specifically investigate damage in fresh tobacco leaves are still scarce. Existing studies indicate that deep learning approaches, particularly the YOLO series, are feasible for detecting damage to fresh tobacco leaves. However, both the detection of plant leaf pests and diseases and the identification of tobacco leaf maturity focus on leaves with intact morphology; their regular geometric features significantly reduce the complexity of detection tasks. In contrast, damage to fresh tobacco leaves presents distinct challenges. Physical damage incurred during mechanical harvesting and transportation frequently results in morphological abnormalities in fresh tobacco leaves, such as irregular tearing, partial defects, and complex wrinkles. These damage features also exhibit dynamic variations as harvesting process parameters change. Unlike common agricultural vision tasks such as disease spot detection, maturity recognition, or leaf counting, fresh tobacco leaf damage detection requires the model to distinguish visually subtle and structurally irregular damage states from normal leaf morphology. In practical harvesting scenes, damaged and intact leaves often share similar color distributions, while folds, veins, shadows, and natural curling may resemble broken regions or missing tissue. This high inter-class similarity and large intra-class variation make the decision boundary between damaged and intact leaves ambiguous. In addition, mechanical harvesting introduces leaf overlap, partial occlusion, non-uniform illumination, motion blur, and scale changes caused by unstable conveying, which further weakens the visibility of discriminative damage cues. These characteristics make the task not only an object detection problem, but also a fine-grained visual state recognition problem under dynamic field conditions. Therefore, an effective model must simultaneously preserve local structural details, integrate multi-scale contextual information, and maintain low computational complexity for real-time deployment on embedded harvesting platforms. To overcome these limitations, deep learning is integrated with image processing to develop an efficient framework for damaged fresh tobacco leaf detection. The proposed approach aims to enable objective evaluation of harvesting quality and real-time detection of mechanically damaged fresh tobacco leaves, thereby reducing reliance on traditional manual inspection. **Figure 1** provides a graphical overview that summarizes the overall workflow of the proposed method. Images of mechanically harvested tobacco leaves were collected under both laboratory and field conditions using two custom-designed acquisition systems. After dataset construction through image preprocessing, YOLOv8n was selected as the baseline model and subsequently improved to enhance detection performance. Comparative experiments were then conducted to systematically evaluate the performance of the proposed model against baseline and representative detection approaches. Finally, the optimized model was deployed on an embedded computing platform to further verify its feasibility and real-time performance under practical operating conditions.

**Figure 1:**
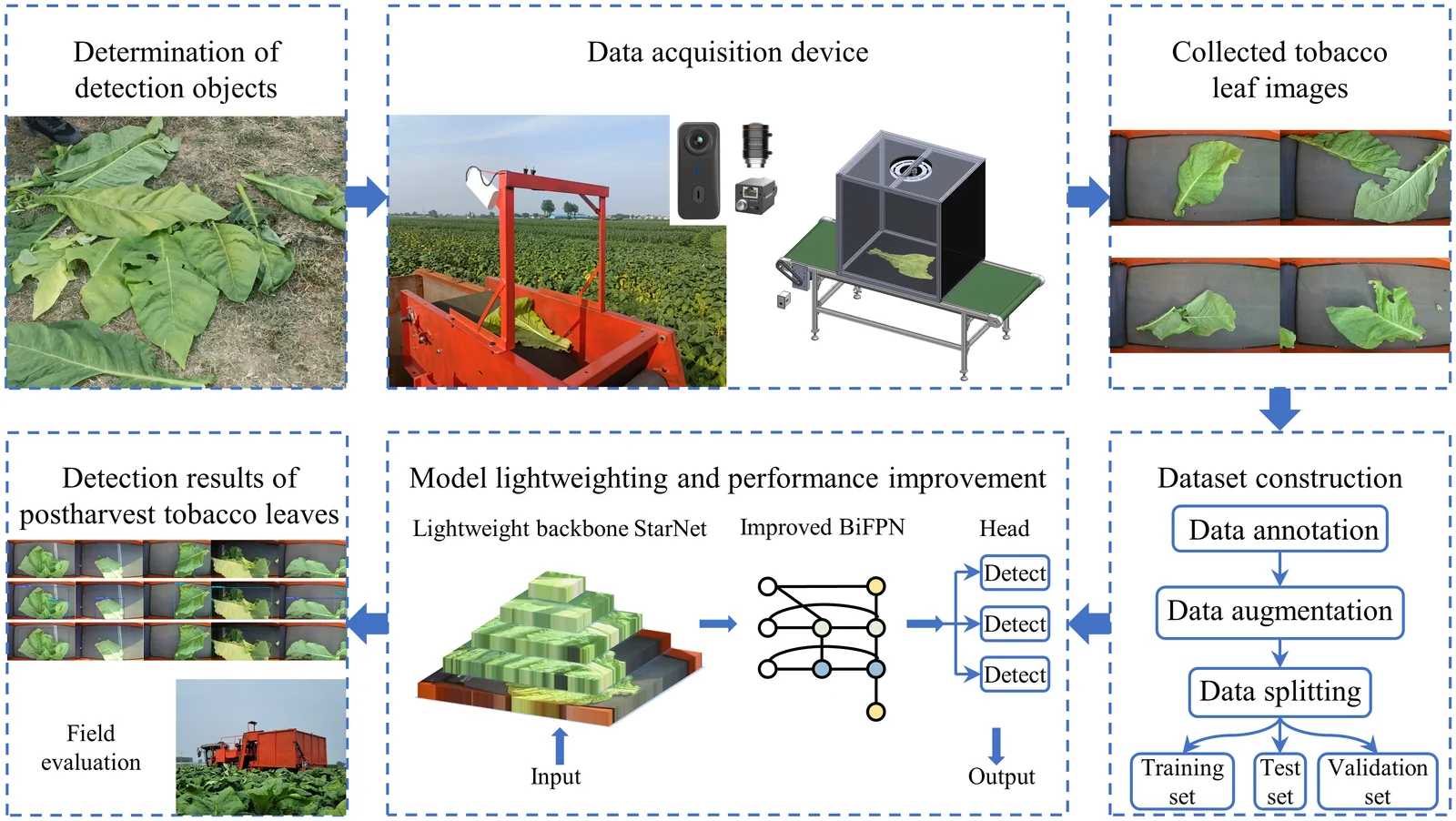
Graphical overview of the study.

## 2 Materials and methods

### 2.1 Dataset construction

#### 2.1.1 Image acquisition

To evaluate the detection of damaged tobacco leaves under different acquisition conditions, a dataset was constructed using tobacco leaf images collected under both laboratory settings and actual harvester operation. Tobacco leaf images under laboratory conditions were acquired by a custom-built image acquisition system, which consists of a conveyor belt, an enclosed dark box, an LED ring light source, an industrial camera, a lens, and a computer. The industrial camera used was an MV-CA016-10GC (Hikvision, China), equipped with an MVL-HF0624M-10MP (Hikvision, China) industrial lens with a focal length of 6 mm. The conveyor belt had a width of 680 mm, while the dark box measured 830 mm in both height and length. As illustrated in **Figure 2**, a pure black stage was employed as the background during image acquisition. This configuration enhanced the visual contrast of damaged regions on tobacco leaves and effectively minimized background interference in subsequent damage detection tasks. All acquired fresh tobacco leaf images were transmitted to and stored on the computer for further processing and dataset construction.

**Figure 2:**
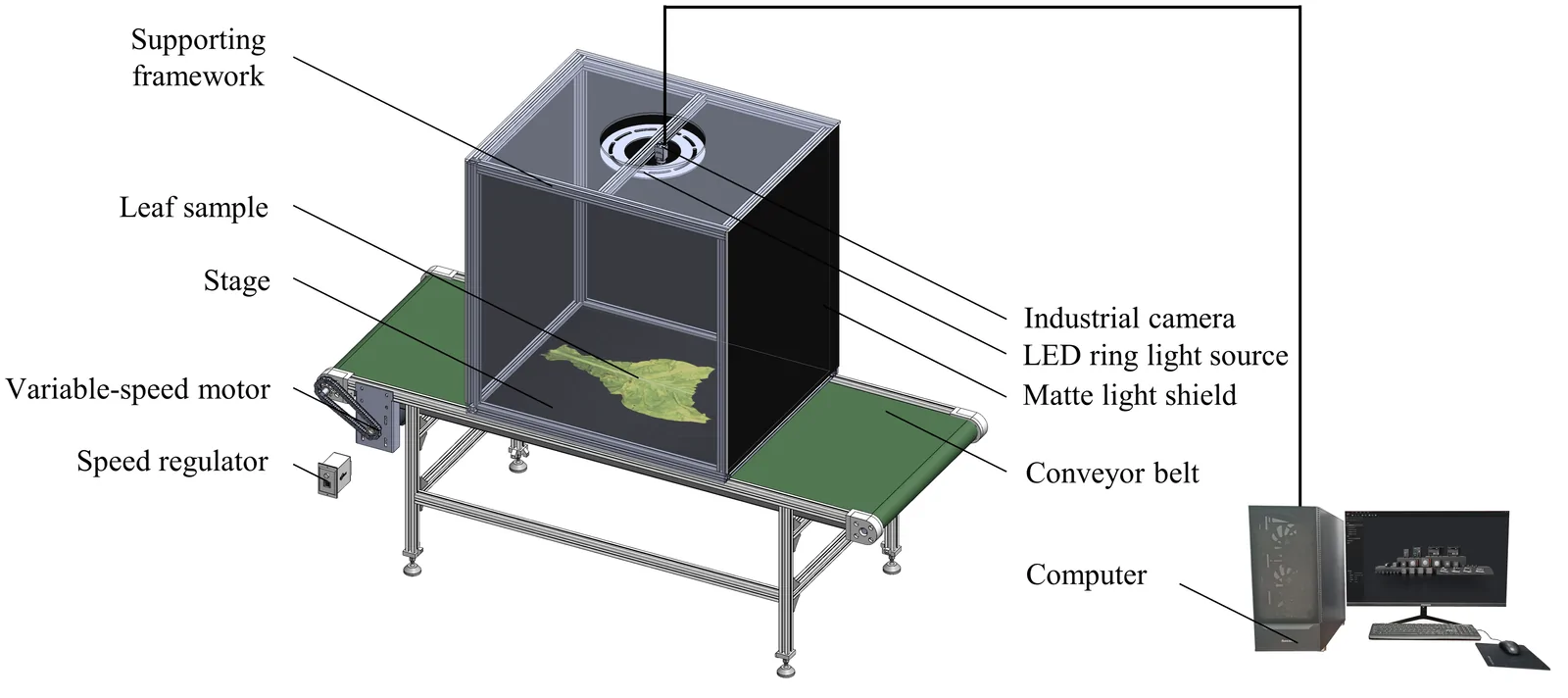
Fresh tobacco leaf image acquisition device in laboratory environment.

For field image acquisition during tobacco harvesting, an image acquisition system is designed based on the 4YZ-2 self-propelled layered tobacco leaf harvester. As shown in **Figure 3**, the system mainly consists of a support frame, a camera mounting plate, a camera, and a mobile power supply. The inclined conveyor belt structure shortens the sliding distance of tobacco leaves during conveying, thereby reducing collisions and friction between leaves and mitigating additional mechanical damage. During harvester operation, the camera mounting plate was aligned parallel to the inclined plane of the conveyor belt to maintain a stable imaging perspective. The camera was installed at a height of 510 mm, ensuring that the entire conveyor belt remained within the field of view. Under this configuration, the acquired images reliably captured the morphological features and damage characteristics of tobacco leaves under actual harvesting conditions.

**Figure 3:**
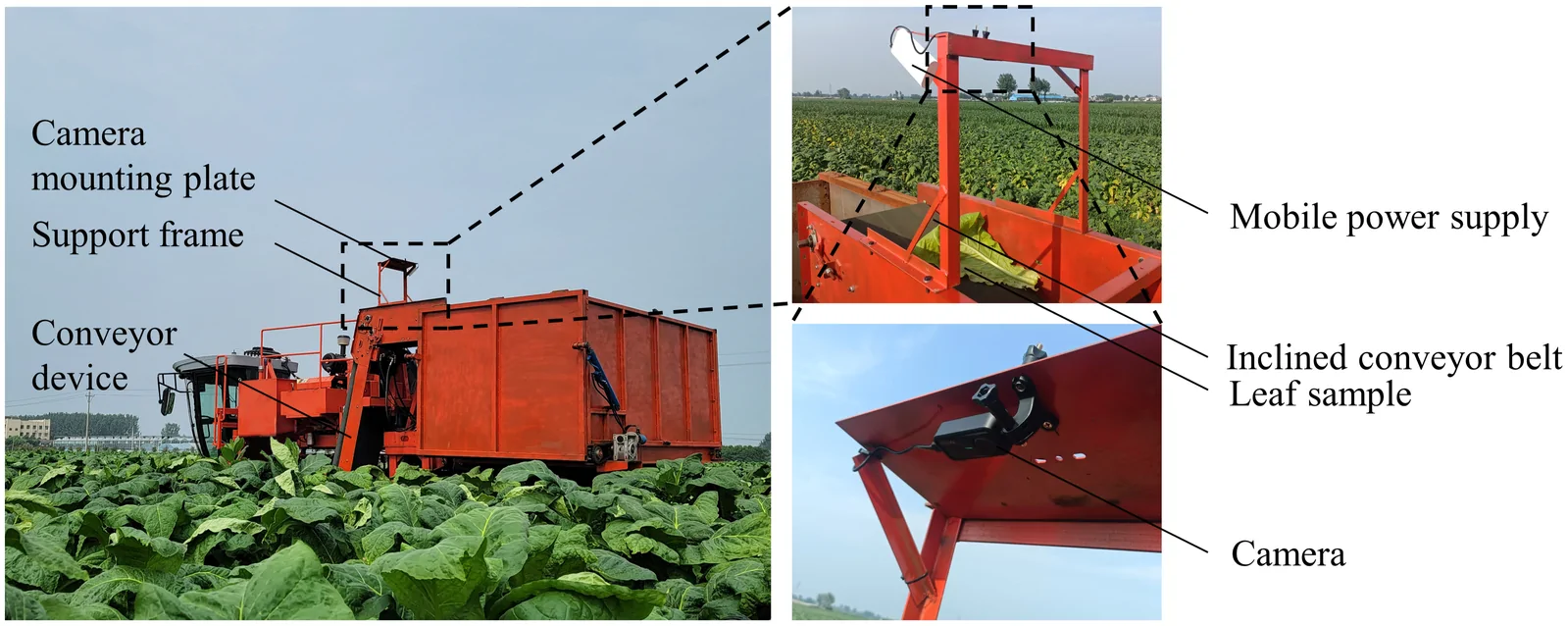
Device and installation position for tobacco leaf image acquisition in field environments.

Tobacco leaf images under natural field conditions were collected at a tobacco planting farm located in Xiangcheng County, Xuchang City, Henan Province, China, from June to September 2024. Video data were recorded using a SARGO G10+ Thumb Action Camera at a resolution of 1920×1080 pixels. To account for variations in illumination and frequent leaf overlap and occlusion during conveyor transport, video sequences were acquired across multiple times of the day and under a range of lighting conditions. This data collection strategy increased the diversity of the target samples for subsequent model training. The harvesting videos were first extracted frame by frame. For each individual leaf passage through the camera field of view, one representative frame in which the tobacco leaf exhibited a relatively complete morphology and could be reliably annotated was retained.

#### 2.1.2 Image annotation and augmentation

A total of 1,321 tobacco leaf images were obtained as the original dataset. According to the operational practice of layered tobacco harvesters, the damage rate is defined, following the standards of the China National Tobacco Corporation, as the ratio of damaged leaves to the total number of leaves collected in the leaf collection box after harvesting. Tobacco leaves with a broken middle part of the main vein or a missing area exceeding one-third of the total leaf area were classified as damaged tobacco leaves, as specified in **Figure 4**.

**Figure 4:**
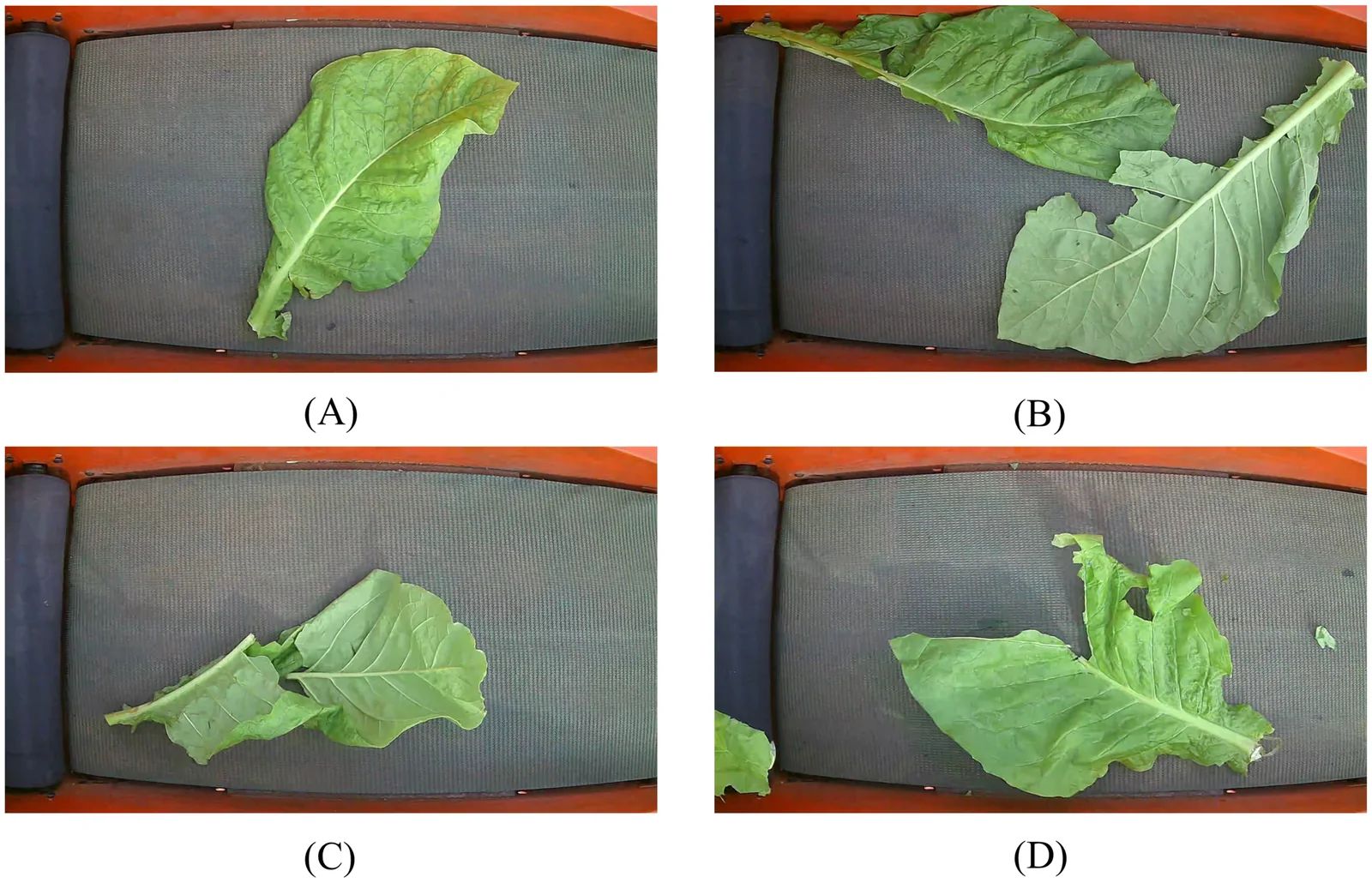
Dataset of mechanically harvested fresh tobacco leaves, **(A)** Intact tobacco leaf, **(B)** Leaves with missing areas exceeding 1/3, **(C)** The main vein broken in the middle, **(D)** The main vein broken in the middle and leaves with missing areas exceeding 1/3.

During dataset construction, only severely blurred frames that prevented reliable identification and annotation were excluded, while visually interpretable frames containing motion blur were retained to preserve the representativeness of practical harvesting conditions. Manual annotations for tobacco leaf images were created in Labelme (https://people.csail.mit.edu/torralba/research/LabelMe_Aina/). In each image, one or more polygonal annotations were used to label intact and damaged tobacco leaves. The original dataset contained 1,321 images containing two categories: intact tobacco leaves and damaged tobacco leaves. The detailed distribution of the original tobacco leaf samples is provided in **Table 1**. Since some images contained both intact and damaged leaves, the image-level category statistics were not mutually exclusive. The annotated dataset was randomly divided into training, validation, and testing subsets at a ratio of 8:1:1 for subsequent model training and evaluation. Only the training subset was subjected to augmentation procedures to enhance sample diversity, including horizontal or vertical flipping, scaling, rotation by ±15°, brightness adjustment, noise addition, and random Gaussian blur. Geometric transformations simulated variations in leaf orientation and viewpoint, brightness adjustment accounted for illumination changes, and noise addition and Gaussian blur represented image quality degradation during mechanical harvesting. Ultimately, the augmented tobacco leaf dataset contained 2,110 images for training, 132 for validation, and 133 for testing. Representative augmented samples are provided in **Figure 5**.

**Table 1:** Distribution of original tobacco leaf samples.

Category	Number of images	Number of instances
Intact leaf	905	968
Damaged leaf	506	543

**Figure 5:**
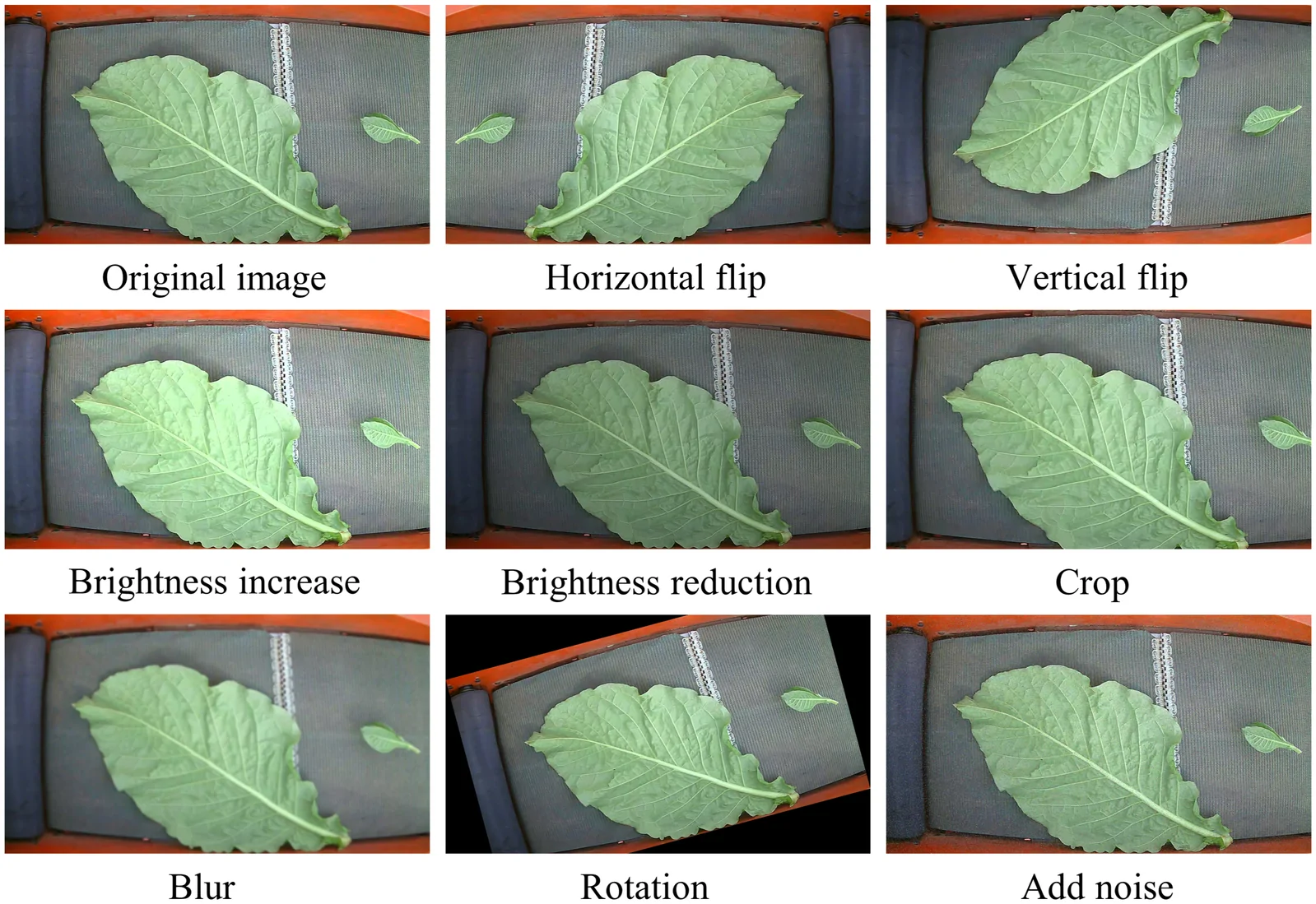
Data augmentation methods.

### 2.2 Baseline model

YOLO has become a widely recognized one-stage detection paradigm in computer vision research. Within the YOLO series, YOLOv8, released by Ultralytics (https://github.com/ultralytics/ultralytics), corresponds to a stable and mature architectural design that has been extensively validated in object detection practice. Among the available variants, YOLOv8n is characterized by a favorable trade-off between detection accuracy and model compactness, making it suitable for deployment on resource-constrained embedded platforms. Accordingly, YOLOv8n served as the baseline framework for damage detection in fresh tobacco leaves, and task-oriented modifications were introduced to address the characteristics of mechanical harvesting scenarios.

The YOLOv8 architecture comprises a backbone, neck, and detection head. The backbone network is responsible for feature extraction and is composed of CBS, C2f, and Spatial Pyramid Pooling-Fast (SPPF) modules. The CBS module integrates Convolution (Conv), BatchNorm2d, and SiLU activation functions to facilitate efficient feature extraction. The C2f module replaces the C3 module used in YOLOv5 (https://github.com/ultralytics/yolov5) and captures rich gradient flow information using the residual structure of the C3 module and the ELAN concept, while having fewer parameters and better feature extraction capabilities. The SPPF module employs spatial pyramid pooling to fuse local and global features, thereby enriching feature representation. The neck serves as an intermediate feature fusion module between the backbone and the detection head. YOLOv8 adopts a neck architecture that integrates the design principles of the Feature Pyramid Network (FPN) and Path Aggregation Network (PAN), enabling effective multi-scale feature fusion. Feature maps from multiple stages of the backbone network are fused to enrich semantic representation and improve localization performance for objects at multiple scales. The head parses target categories and location information from processed features, generating final detection results with Binary Cross-Entropy (BCE) loss for classification and a combination of Distribution Focal Loss (DFL) and Complete Intersection over Union (CIoU) loss for regression. CIoU loss is employed to refine bounding box regression by jointly considering overlap area, center distance, and aspect ratio consistency. The original YOLOv8n architecture is illustrated in **Figure 6**.

**Figure 6:**
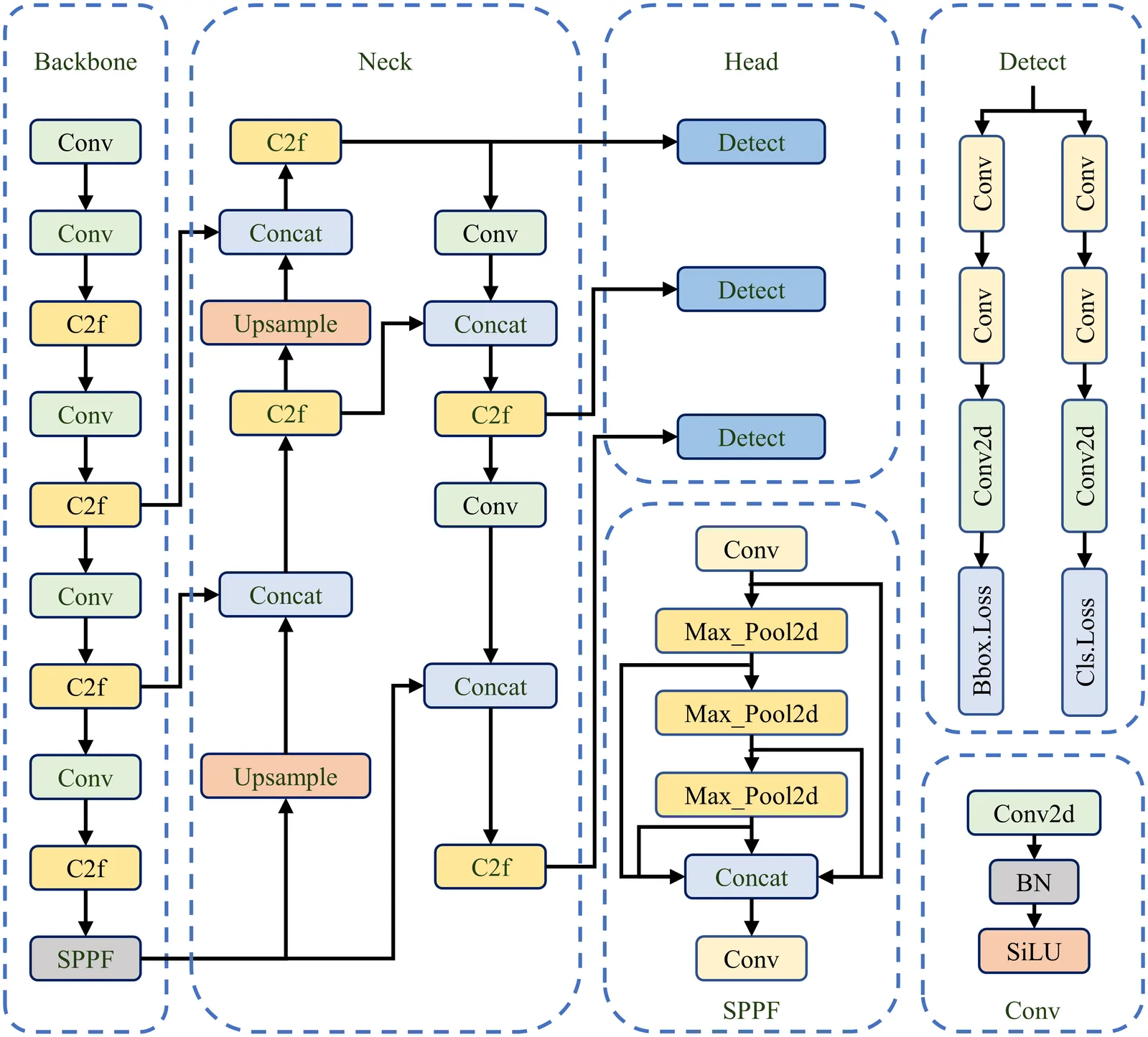
The network structure diagram of the original YOLOv8n.

### 2.3 DTL-YOLO network architecture

The adjustment of harvesting components and the unstable conveying state of the layered tobacco harvester pose considerable challenges to real-time quality evaluation of fresh tobacco leaves. During mechanical harvesting and transport, tobacco leaves may exhibit overlap, partial occlusion, motion blur, non-uniform illumination, and irregular deformation. Moreover, mechanically induced damage is not characterized by a fixed visual pattern; midrib fracture, partial tissue loss, tearing, and severe wrinkles may appear at different locations and scales, while some normal folds or shadows can resemble damaged regions. Therefore, the detector must preserve fine local structural cues, integrate multi-scale contextual information, and maintain low computational complexity for real-time embedded deployment. To address these requirements, YOLOv8n was selected as the baseline framework and improved in terms of backbone representation, neck feature fusion, and downsampling information preservation. Specifically, StarNet was adopted to replace the original YOLOv8n backbone, thereby reducing model complexity while maintaining efficient feature extraction (Lu et al., 2025). Subsequently, the neck component was redesigned using an improved Bidirectional Feature Pyramid Network (BiFPN), enabling more effective multi-scale feature interaction for damaged leaves with different sizes, deformation patterns, and occlusion degrees. In addition, the lightweight ADown module derived from YOLOv9 was employed to replace selected standard convolutional downsampling operations within the improved BiFPN, alleviating the loss of discriminative structural information while maintaining computational efficiency. The improved YOLOv8n network was renamed DTL-YOLO (Damaged Tobacco Leaf-YOLO). The optimized network model is shown in **Figure 7**.

**Figure 7:**
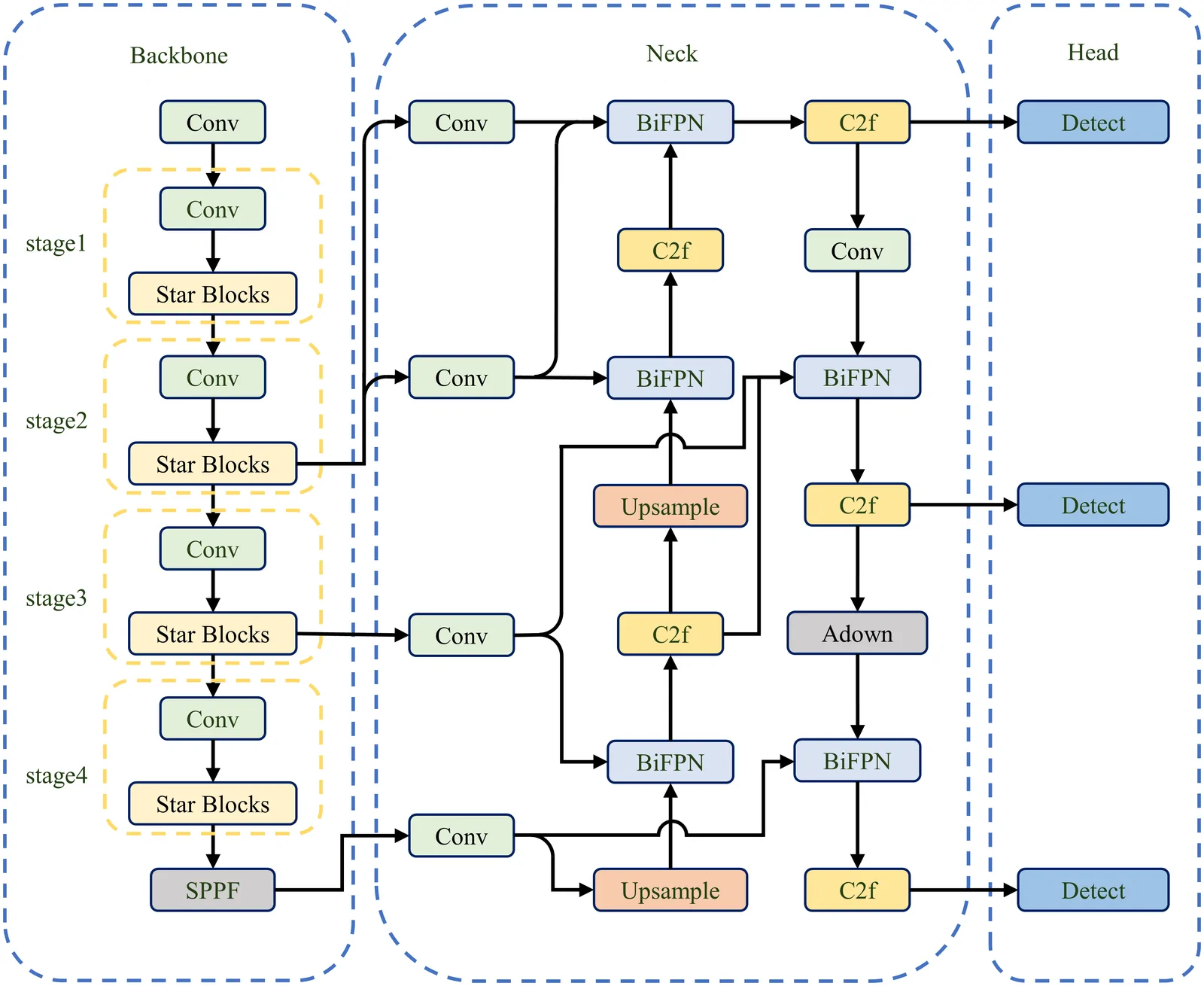
The network structure diagram of the DTL-YOLO.

#### 2.3.1 StarNet lightweight backbone network

Ma et al. (2024) introduced StarNet as a network framework that prioritizes computational efficiency through lightweight architectural design, enabling effective feature representation under constrained resources. Compared with conventional lightweight network structures, StarNet achieves competitive performance without relying on complex architectural designs or fine-tuned hyperparameters. It features a four-stage hierarchical architecture, in which each stage directly uses convolutional layers for downsampling and performs feature extraction through Star Blocks, making it well suited for use as a compact and efficient backbone network. For efficiency, layer normalization is replaced with batch normalization, which is applied after the depthwise convolution. A core component of StarNet is the Star Operation, which fuses features from different subspaces via element-wise multiplication. The Star Operation has the ability to map inputs to a feature space with extremely high-dimensional non-linearity. After multiple layers, it can significantly increase the hidden dimension, nearly reaching an infinite dimension. Owing to this property, the Star Operation can provide more effective feature fusion than the summation operation, despite its simplicity. The Star Operation is shown in **Figure 8**. Integrating StarNet into the YOLOv8 backbone makes the network structure more compact and enables it to achieve satisfactory performance under limited computational resources.

**Figure 8:**
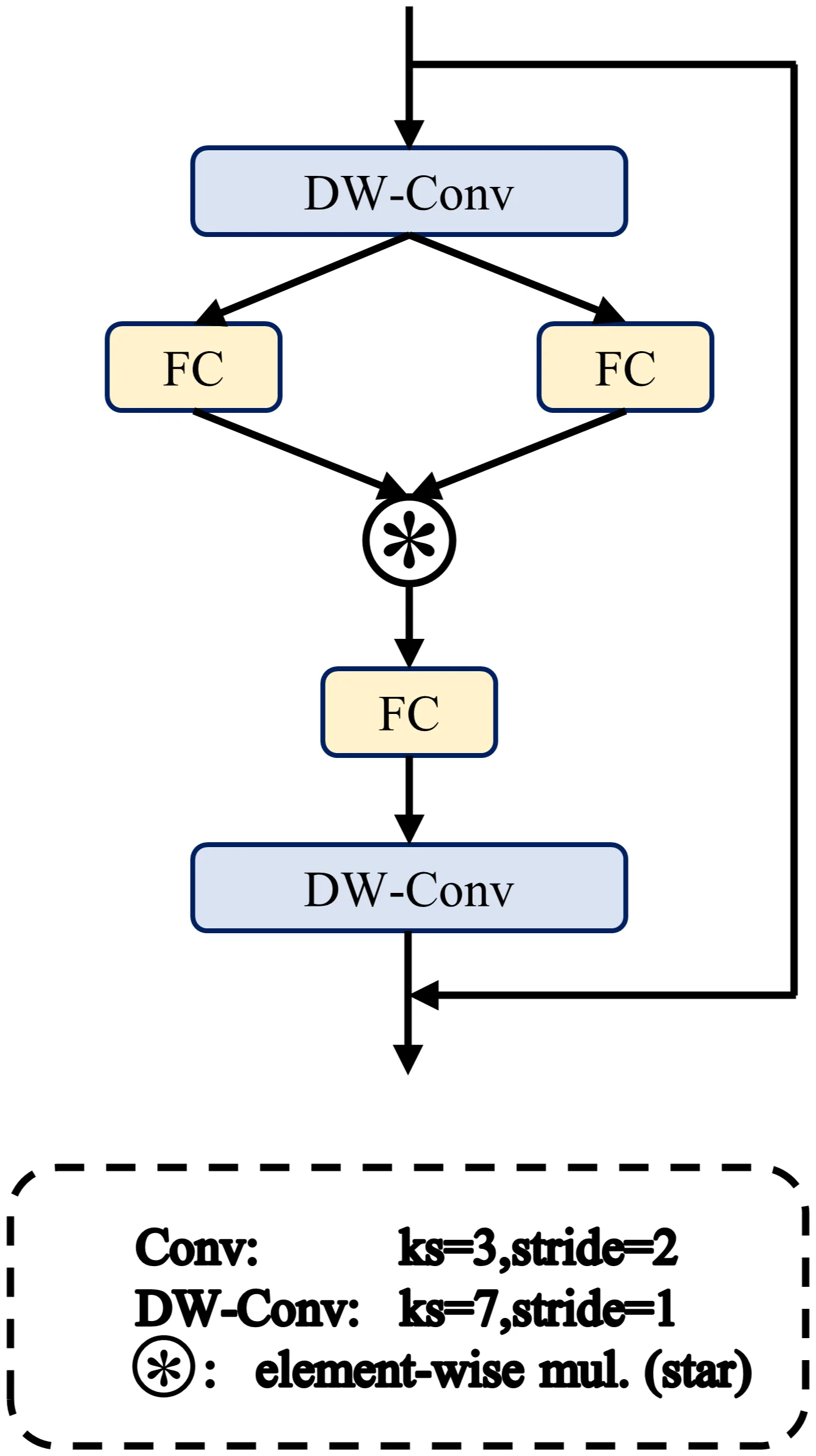
The structure of Star Block.

#### 2.3.2 Improved neck network: Bidirectional Feature Pyramid Network (BiFPN)

In the collected images of fresh tobacco leaves, damage exhibits substantial variation in severity, missing location, and scale, including differences between midrib breakage occurring in the central region of the leaf and partial leaf loss. To accommodate these characteristics, the original neck structure of the network was replaced with an improved Bidirectional Feature Pyramid Network (BiFPN) (Tan et al., 2020), which enables more effective integration of shallow spatial details and deep semantic information. This design strengthens the representation of both local structural details and global leaf characteristics, thereby improving the identification of damage regions with different visual scales and degrees of occlusion. Owing to the unidirectional information flow in traditional Feature Pyramid Networks (FPN) (**Figure 9A**), the incorporation of detailed information from shallow layers into deep layer representations is limited, which commonly results in the loss of low level features. The Path Aggregation Network (PANet) addresses this issue by introducing an additional bottom-up pathway to enhance information flow and gradient propagation (**Figure 9B**). However, the single-input nodes in PANet contribute little to effective feature fusion while increasing model parameters and computational cost. To improve efficiency, BiFPN removes these single-input edges and introduces learnable weight parameters to adaptively balance contributions from different feature levels. Each bidirectional pathway is implemented as an individual feature-processing layer, within which alternating top-down and bottom-up operations facilitate enhanced feature fusion. Unlike FPN and PANet, which rely solely on lateral connections to combine top-down and bottom-up pathways, BiFPN supports cross-level skip connections, further enhancing the efficiency and flexibility of multi-scale feature integration.

**Figure 9:**
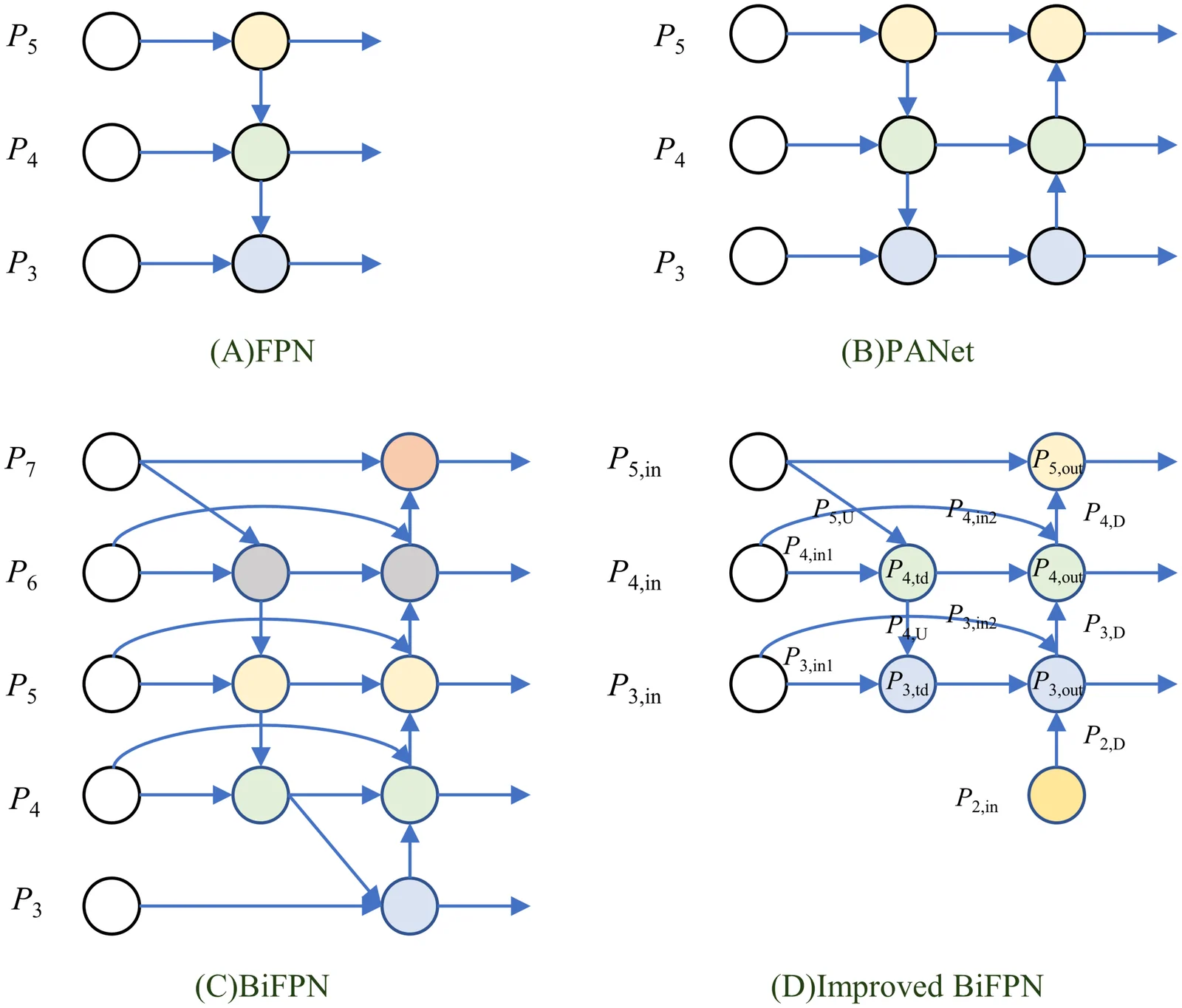
Different neck networks.

The original BiFPN consists of five input feature levels, as illustrated in **Figure 9C**. The neck network of YOLOv8 typically incorporates 3 input feature layers of varying sizes from the backbone network, specifically Pyramid Level 5 (*P*_5_), Pyramid Level 4 (*P*_4_), and Pyramid Level 3 (*P*_3_). This configuration is generally sufficient for detecting objects at relatively large spatial scales. However, for features requiring fine-grained interpretation such as midrib fractures occurring in the central region of fresh tobacco leaves, the spatial resolution of these three feature layers may be insufficient. Therefore, DTL-YOLO introduces a four-level input design by extending feature fusion down to Pyramid Level 2 (*P*_2_), allowing shallow high-resolution features to provide additional spatial details for subtle damage recognition. To further enhance feature quality, a CBS module is applied before each skip connection to strengthen the features at each fusion node, thus boosting the model’s ability to represent features and its sensitivity to subtle detail variations (**Figure 9D**).

#### 2.3.3 Lightweight downsampling module ADown

The downsampling module helps the model capture higher-level features while reducing the spatial dimensions of feature maps and lowering computational cost. In this study, the ADown module (Wang C-Y. et al., 2024) was integrated into the improved BiFPN neck of YOLOv8 to replace conventional downsampling operations, facilitating more effective modeling of the global structural characteristics of fresh tobacco leaf images. The structure of the ADown module is illustrated in **Figure 10**. The ADown module combines Average Pooling and Max Pooling to effectively balance feature compression and information loss while reducing computational cost. Specifically, an average pooling operation is first applied to the input feature map to reduce its spatial dimensions and obtain a global average representation. The feature map is then divided into two branches, each receiving the same number of channels.

**Figure 10:**
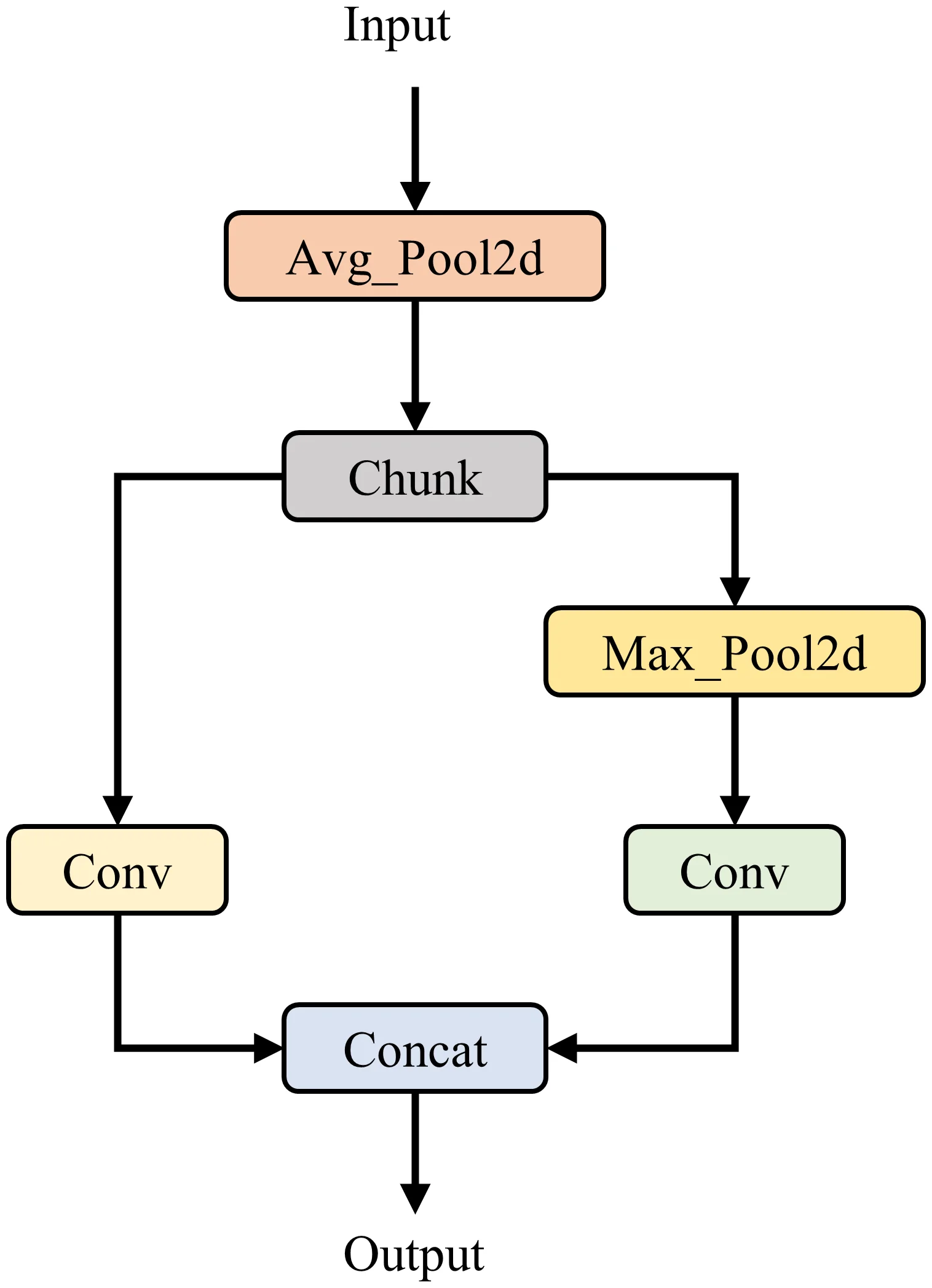
The structure of ADown module.

One branch is processed using a convolution operation with a 3×3 kernel. For the other branch, the feature map is first subjected to a Max Pooling operation, resulting in a twofold reduction in both height and width, preserving the most prominent and critical feature information in local regions. Following this step, channel wise feature integration is performed using a 1×1 convolution, enabling better capture of correlations between channels. The outputs of the two branches are ultimately combined by means of a Concat operation.

### 2.4 Experimental settings and training configuration

All experiments were performed on the same self-collected fresh tobacco leaf dataset under identical training settings and a consistent hardware/software environment. Experiments were performed on a workstation equipped with Windows 11 (64-bit), 32 GB RAM, an Intel Core i7-12700KF CPU, and an NVIDIA GeForce RTX 3080 Ti graphics card with 12 GB VRAM. The implementation was based on PyTorch 2.5.0 with Python 3.9.0. PyTorch was installed with the CUDA 12.1 build (cu121), and the code was developed using PyCharm IDE.

The training configuration employed a batch size of 16, with the initial learning rate set to 0.01 and the momentum set to 0.937. A fixed random seed was used during training to improve experimental reproducibility. Model parameters were optimized using Stochastic Gradient Descent (SGD) for 300 epochs. Model training was performed from scratch, with no reliance on pre-trained weights for parameter initialization.

### 2.5 Evaluation metrics for model performance

To provide a comprehensive evaluation of model performance, this study uses precision, recall, mean Average Precision (mAP), and model size as evaluation metrics. Precision refers to the ratio of true positives (TP) to all predicted positive samples (TP+false positives (FP)), while recall is the ratio of true positives (TP) to all actual positive samples (TP+false negatives (FN)). Under a specified Intersection over Union (IoU) threshold, Average Precision (AP) is derived from the corresponding precision-recall (PR) curve. Accordingly, mAP_50_ is obtained by averaging AP over the two classes (intact and damaged leaves) at IoU=0.5, whereas mAP_50–95_ is calculated by averaging the AP values across multiple IoU thresholds ranging from 0.50 to 0.95 at intervals of 0.05, followed by averaging over all categories. Model size characterizes the storage footprint of the final trained weight files, and compact models are therefore more appropriate for use in resource-limited mobile or embedded deployment scenarios. These metrics are calculated as shown in Equations 1–4:

(1) Precision = TP/TP+FP)×100%

(2) Recall = TP/(TP+FN)×100%

(3) $$AP=\;\int_{0}^{1}P\left(R\right)dR\times 100\%$$

(4) $$mAP=\frac{1}{n}\sum_{i=1}^{n}AP_{i}\times 100\%$$

For the two classes (intact and damaged leaves), TP, FP, and FN were counted on a per-class basis under a one-to-one matching rule at the specified IoU threshold. For a given class, a prediction was counted as a true positive if it matched an unmatched ground-truth instance of the same class with IoU greater than or equal to the threshold; otherwise, it was counted as a false positive (including unmatched detections, duplicate detections, or incorrect class assignments). Ground-truth instances that were not matched by any prediction of the same class were counted as false negatives. Alongside the standard detection accuracy measures, floating-point operations (FLOPs) and the number of parameters characterize computational complexity and resource requirements, while inference speed is measured in frames per second (FPS).

## 3 Results and discussion

### 3.1 Comparative experiments

#### 3.1.1 Comparison of StarNet with other lightweight backbones

Deep learning-based object detection algorithms provide a feasible approach for damage detection of fresh tobacco leaves. Nevertheless, the limited computing power of mobile and edge devices imposes constraints on the practical application of tobacco leaf damage detection. In this study, fresh tobacco leaves are presented at a largely consistent spatial scale in the acquired images, while the visual appearance of midrib breakage in damaged leaves remains highly similar to that of intact leaves. This enables an moderate reduction in network scale while maintaining detection performance. Moreover, typical agricultural application scenarios often require that network models be deployed on mobile devices or embedded systems. Thus, reducing redundant computations of the model while preserving detection accuracy is a primary objective of this research. Consistent with the Star Operation in StarNet, a simple network has been shown to achieve competitive performance with a simpler design. **Table 2** summarizes the detection performance when replacing the original backbone of the YOLOv8n network with different lightweight backbones.

**Table 2:** Performance comparison of lightweight backbone replacements in YOLOv8n.

Backbone	Precision/%	Recall/%	mAP_50_/%	mAP_50-95_/%	Params/M	FLOPs/G	Size/MB
MobileNetV4	72.2	**85.3**	85.0	79.3	5.7	22.5	11.7
FasterNet	**85.5**	78.4	**86.5**	**82.0**	4.2	10.7	8.6
EfficientViT	65.3	82.0	80.4	75.6	4.0	9.4	8.8
MambaOut	71.0	80.3	84.5	77.1	9.24	24.0	18.7
**StarNet**	73.9	78.9	84.4	78.9	**2.21**	**6.5**	**4.7**
GhostHGNetV2	74.0	78.8	82.4	77.9	2.31	6.8	4.9
HGNetV2	83.8	71.4	84.8	79.4	2.35	6.9	5.0
LSKNet	77.2	75.3	82.7	74.8	5.89	19.7	12.2

Bold values indicate the best performance among all compared methods.

Compared with other lightweight backbone networks, StarNet exhibits an effective balance between detect performance and computational cost on the mechanically harvested fresh tobacco leaf dataset. MobileNetV4 (Qin et al., 2024), MambaOut (Yu and Wang, 2024), and FasterNet (Chen et al., 2023a) yield slightly higher mAP_50_, but require larger parameter counts and model size. EfficientViT (Liu et al., 2023) and LSKNet (Li et al., 2023a) show lower detection accuracy while incurring higher computational cost in terms of parameter counts and FLOPs. In contrast to GhostHGNetV2 (Tang et al., 2022) and HGNetV2 (Zhao et al., 2024), StarNet achieves the smallest parameter count, FLOPs, and model size. StarNet attains an mAP_50–95_ of 78.9%, which is 0.5 and 0.4 percentage points lower than HGNetV2 (79.4%) and MobileNetV4 (79.3%), respectively, and 3.1 percentage points lower than FasterNet (82.0%). Relative to HGNetV2, StarNet also reduces computation by 0.4 GFLOPs. Given the deployment-oriented objective of this study, the modest reduction in mAP_50–95_ is considered an acceptable trade-off for the substantial savings in computation and model size. Accordingly, StarNet was selected as the backbone for subsequent experiments.

#### 3.1.2 Effect of ADown position on detection performance

In this study, the ADown module was introduced to replace standard convolution-based downsampling operations within the downsampling transitions of the improved BiFPN. Although ADown can be deployed at different downsampling stages, performance varied across replacement positions. The experimental evaluation reveals that the introduction of the ADown module at the *P*_4_-to-*P*_5_ feature map conversion stage resulted in the best performance. As shown in **Table 3**, implementing the ADown module at this specific position achieves improved performance, with Precision and mAP_50_ increasing by 4.1 and 5.0 percentage points, respectively, compared with the scenario where no replacement is made. A plausible explanation is that downsampling at the *P*_4_-to-*P*_5_ transition affects high-level feature representations that are critical for large leaf regions, and stride-based convolutional downsampling may discard fine boundary or texture cues during this step. By combining average-pooling and max-pooling branches, ADown can preserve salient responses while compressing spatial resolution, which benefits the representation of large-area tobacco leaf targets in images. In addition, this replacement improves the selectivity of feature representations by enhancing informative cues and suppressing less relevant responses, without materially increasing computational overhead, thereby supporting deployment-oriented use.

**Table 3:** Performance comparison of different ADown insertion configurations in the improved BiFPN neck.

Configuration	A	B	C	Precision/%	Recall/%	mAP_50_/%	mAP_50-95_/%	FLOPs/G
Exp. A	✓			**83.6**	73.9	84.1	79.2	5.3
Exp. B		✓		77.7	82.5	84.6	79.8	5.3
**Exp. C**			✓	80.9	81.0	**88.4**	**82.8**	5.4
Exp. D	✓	✓		72.9	84.2	84.3	78.4	**5.2**
Exp. E		✓	✓	74.3	80.8	84.7	79.7	5.3
Exp. F	✓		✓	68.5	**89.0**	84.7	77.5	5.3
Exp. G	✓	✓	✓	75.5	80.8	84.7	78.9	**5.2**

A, B, and C correspond to the downsampling transitions *P*_2_-to-*P*_3_, *P*_3_-to-*P*_4_, and *P*_4_-to-*P*_5_, respectively. Bold values indicate the best performance among all compared methods.

To illustrate the differences before and after model optimization and to examine how the network attends to discriminative regions, feature response maps corresponding to different ADown insertion positions were visualized using Gradient-weighted Class Activation Mapping (Grad-CAM). Grad-CAM generates class-discriminative heatmaps by weighting activation maps with their corresponding gradients, thereby emphasizing spatial regions that exert the greatest influence on the model’s predictions. The resulting visualizations are presented in **Figure 11**.

**Figure 11:**
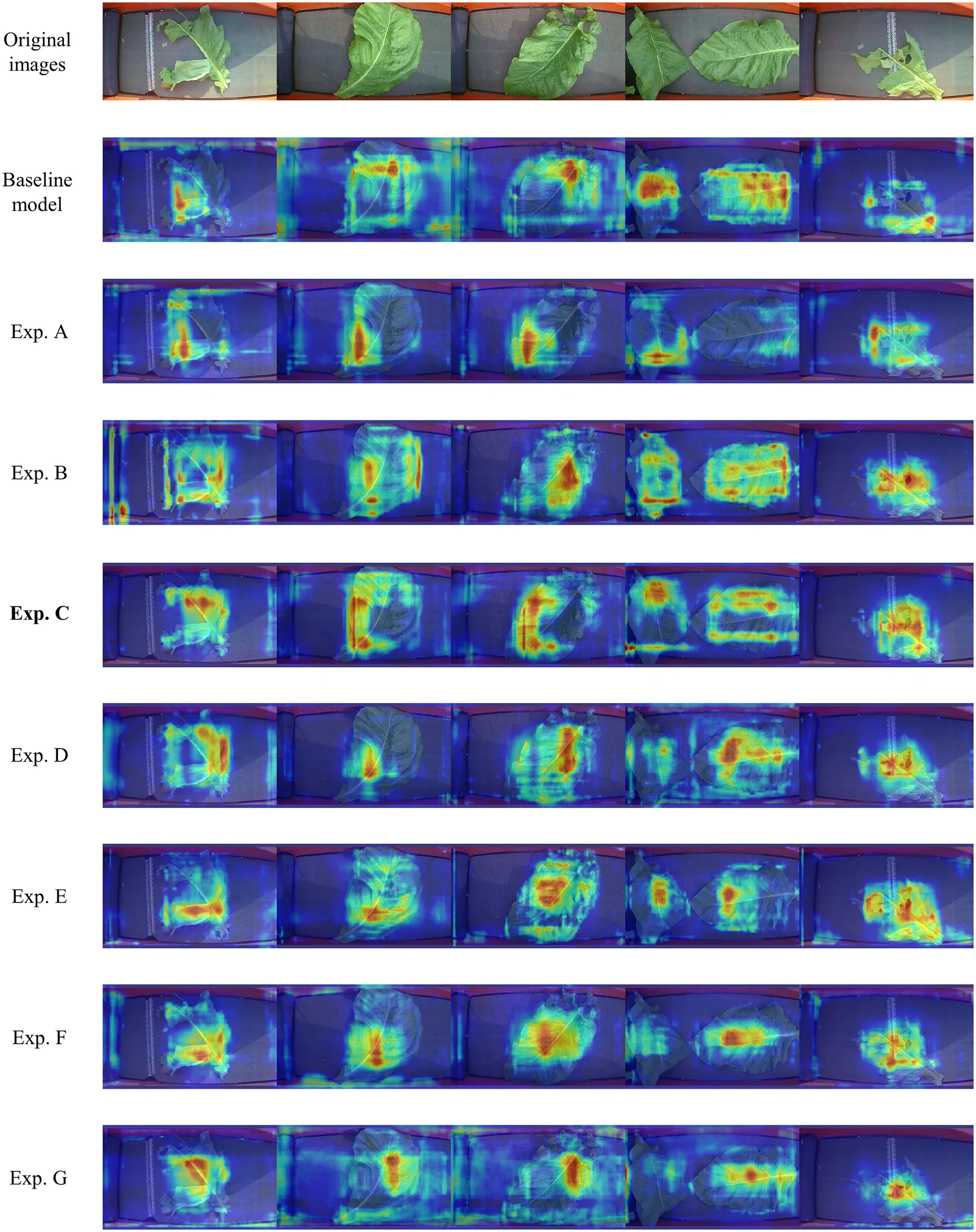
Grad-CAM visualization of different ADown insertion configurations in the improved BiFPN neck.

#### 3.1.3 Comparison of downsampling modules

To assess the effectiveness of the ADown module, six representative lightweight downsampling designs were selected for comparison: ADown, a novel lightweight downsampling module introduced in YOLOv9; SPDConv (Sunkara and Luo, 2022) and LDConv (Zhang et al., 2024), two representative depthwise separable convolution-based downsampling methods; V7DownSampling, the proprietary downsampling module from YOLOv7 (Wang et al., 2023a); Wavelet pooling (Williams and Li, 2018), a transform-domain downsampling approach and Context Guided Block (CGB) down-sampling (Wu et al., 2021), a context-aware downsampling strategy. **Table 4** reports the effects of these lightweight downsampling modules on detection performance in the enhanced network, with all experiments conducted under identical training configurations to ensure comparability and reproducibility.

**Table 4:** Performance comparison of lightweight downsampling modules integrated into the proposed network on the self-collected tobacco leaf dataset.

Downsampling module	Precision/%	mAP_50_/%	mAP_50-95_/%	Params/M	FLOPs/G
SPDConv	79.1	83.4	78.7	1.30	5.5
LDConv	79.9	85.5	80.5	1.19	5.4
Wavelet Pooling	**88.0**	86.0	80.8	1.17	5.4
**ADown**	80.9	**88.4**	**82.8**	**1.16**	5.4
CGBlock_Down	75.1	81.6	76.5	1.28	5.5
V7DownSampling	74.0	83.6	78.2	1.17	5.4

Bold values indicate the best performance among all compared methods.

Among the six downsampling operators evaluated in the improved BiFPN neck, the model using ADown achieved the best overall accuracy, with mAP_50_ of 88.4% and mAP_50–95_ of 82.8%. At comparable FLOPs, the Wavelet Pooling operator yielded higher precision than ADown, LDConv, and V7DownSampling, and it also had a lower parameter count among these four operators. This gain in precision suggests fewer false positives, but it did not translate into improvements in mAP_50_ or mAP_50-95_. ADown outperformed Wavelet Pooling by 2.4 percentage points in mAP_50_ and by 2.0 percentage points in mAP_50-95_. By contrast, SPDConv and the Context Guided Block downsampling module produced lower mAP_50_ while incurring higher FLOPs and larger parameter counts, making them less suitable for lightweight deployment. The model incorporating the ADown operator achieved a parameter count of 1.16M and a computational cost of 5.4 GFLOPs. Taken together, the ADown-based model delivered the best accuracy-efficiency balance among the compared downsampling operators for fresh tobacco leaf damage detection.

### 3.2 Ablation experiment

Ablation experiments combining individual module evaluation and progressive module integration were conducted to evaluate the effects of the proposed modifications on detection performance and model complexity. The corresponding configurations and experimental results are presented in **Table 5**. As shown in **Table 5**, replacing the original backbone with StarNet substantially reduced the computational requirements, although the detection metrics decreased to some extent. Replacing the original neck with the improved BiFPN further reduced model complexity and increased recall, while precision, mAP_50_, and mAP_50–95_ decreased. These results indicate that the two lightweight modifications mainly contributed to reducing model complexity, although some loss in detection performance was introduced. When StarNet and the improved BiFPN were combined, the parameter count, FLOPs, and model size were further reduced to 1.19 M, 5.4 G, and 2.6 MB, respectively. The subsequent introduction of ADown at the *P*_4_-to-*P*_5_ transition improved all four detection metrics without increasing computational complexity, thereby recovering part of the detection performance lost during the lightweight modification.

**Table 5:** Ablation study of the proposed improvements to YOLOv8n.

YOLOv8n	StarNet	Improved BiFPN	ADown	Precision/%	Recall/%	mAP_50_/%	mAP_50-95_/%	Params/M	FLOPs/G	Size/MB
✓				**83.7**	79.1	86.6	82.7	3.01	8.1	6.3
✓	✓			73.9	78.9	84.4	78.9	2.21	6.5	4.7
✓		✓		76.1	**81.5**	85.3	80.2	1.99	7.1	4.2
✓	✓	✓		76.8	78.9	83.4	79.3	1.19	5.4	**2.6**
✓	✓	✓	✓	80.9	81.0	**88.4**	**82.8**	**1.17**	**5.4**	**2.6**

Bold values indicate the best performance among all compared methods.

Compared with the original YOLOv8n, the proposed model showed a 2.8 percentage point decrease in precision and a 1.9 percentage point increase in recall, while mAP_50_ and mAP_50–95_ increased by 1.8 and 0.1 percentage points, respectively. Meanwhile, the number of parameters, FLOPs, and model size were reduced by 61.1%, 33.3%, and 58.7%, respectively. Overall, DTL-YOLO achieves a favorable balance between detection performance and computational efficiency compared with YOLOv8n.

### 3.3 Comparative experiments and analysis of different models

To assess the overall performance of DTL-YOLO for fresh tobacco leaf damage detection, the proposed model was compared with representative lightweight one-stage detectors, including YOLOv5s, YOLOv5n, YOLOv8s, YOLOv9t (Wang C-Y. et al., 2024), and YOLOv10n (Wang A. et al., 2024). The comparative results are reported in **Table 6**.

**Table 6:** Performance comparison of different models for fresh tobacco leaf damage detection.

Model	Precision/%	Recall/%	mAP_50_/%	mAP_50-95_/%	Param/M	FLOPs/G	FPS
YOLOv5s	82.1	80.0	86.8	81.9	9.11	23.8	126
YOLOv5n	81.4	79.4	86.4	81.9	2.50	7.1	130
YOLOv8n	83.7	79.1	86.6	82.7	3.01	8.1	143
YOLOv8s	**84.3**	78.6	87.4	82.4	11.1	28.4	121
YOLOv9t	71.3	80.7	79.8	75.4	2.0	7.6	89
YOLOv10n	79.5	72.5	80.9	76.6	2.27	6.5	127
**DTL-YOLO**	80.9	**81.0**	**88.4**	**82.8**	**1.17**	**5.4**	**168**

Bold values indicate the best performance among all compared methods.

As shown in **Table 6**, YOLOv5n, YOLOv9t, and YOLOv10n are more lightweight than YOLOv8n in terms of parameters and FLOPs, but they achieve lower precision and mAP metrics. Additionally, YOLOv8n achieves the highest FPS, suggesting a stronger real-time capability than YOLOv5n, YOLOv9t, and YOLOv10n under the same evaluation settings. By contrast, YOLOv5s and YOLOv8s achieve higher accuracy metrics, but their larger parameter counts and FLOPs make them less favorable when lightweight deployment is a primary constraint. Overall, DTL-YOLO achieves the highest recall and mAP metrics among the compared models, while also requiring the fewest parameters and the lowest FLOPs.

Specifically, relative to YOLOv5s, YOLOv5n, YOLOv8s, YOLOv9t, and YOLOv10n, DTL-YOLO reduces parameter counts by 87.16%, 53.20%, 89.46%, 41.50%, and 48.46%, lowers FLOPs by 77.31%, 23.94%, 80.99%, 28.95%, and 16.92%, and increases mAP_50_ by 1.6, 2.0, 1.0, 8.6, and 7.5 percentage points. These comparisons show that DTL-YOLO achieves a favorable accuracy-efficiency trade-off, reducing computational cost while maintaining competitive detection performance, which supports its suitability for real-time deployment in harvesting scenarios.

To further analyze the detection performance of different categories, per-class results of YOLOv8n and DTL-YOLO are presented in **Table 7**. DTL-YOLO achieved the same AP_50_ for intact tobacco leaves as YOLOv8n and improved the AP_50_ and recall of damaged tobacco leaves by 3.5 and 5.5 percentage points, respectively. These results indicate that the proposed lightweight model maintained category-level detection performance while enhancing the recognition of damaged leaves.

**Table 7:** Per-class detection performance comparison between YOLOv8n and DTL-YOLO.

Model	Category	Precision/%	Recall/%	AP_50_/%
YOLOv8n	Intact tobacco leaf	86.9	87.4	91.5
	Damaged tobacco leaf	80.5	70.9	81.8
DTL-YOLO	Intact tobacco leaf	86.2	85.6	91.5
	Damaged tobacco leaf	75.6	76.4	85.3

### 3.4 Visualization and analysis of prediction results

Prediction results were visualized to qualitatively assess detection performance by illustrating representative true-positive (TP), false-positive (FP), and false-negative (FN) cases. As shown in **Figure 12**, color-coded bounding boxes denote the three outcomes: green indicates correctly detected fresh tobacco leaves (TP), blue indicates false detections (FP), and red indicates missed detections (FN). Under harvesting conditions, leaf congestion and occlusion can occur when the picking mechanism is improperly adjusted or when conveying is delayed or unstable, leading to leaf stacking, increased overlap, and frequent occlusions. In such scenarios, errors in object localization and detection reliability, including missed targets and false positives, become more prevalent. As shown in **Figures 12A, E**, DTL-YOLO reduces missed detections in these challenging cases, thereby supporting more reliable quality assessment of fresh tobacco leaves during mechanical harvesting. In addition, the improved model reduces false detections under harvesting conditions. **Figures 12B–D** further show that DTL-YOLO provides more reliable localization and classification for leaves exhibiting midrib fractures and missing regions, and it remains effective even when targets are partially occluded. These examples highlight the robustness of DTL-YOLO to leaf overlap and occlusion under complex field operating conditions.

**Figure 12:**
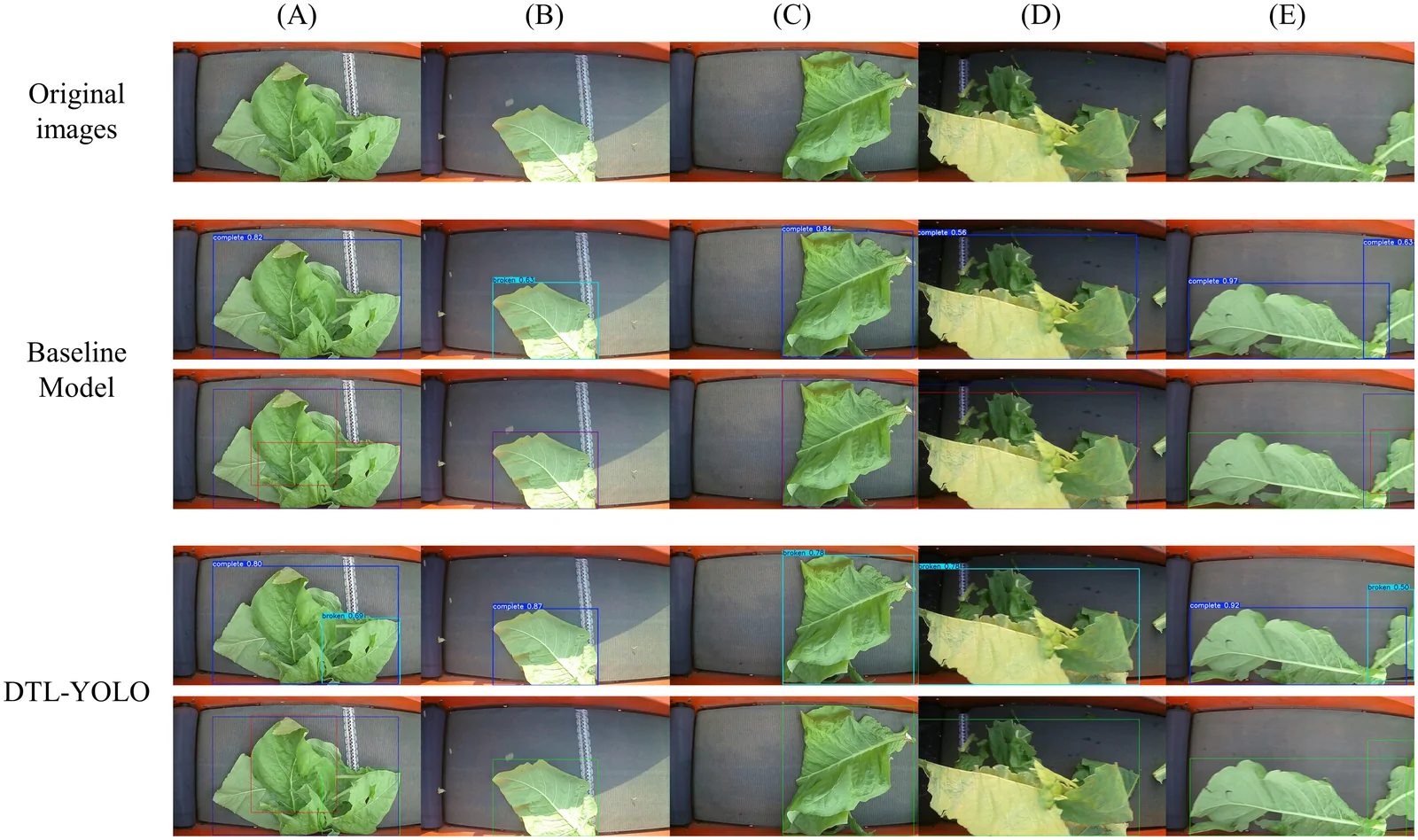
Visual comparison of detection results between YOLOv8n and DTL-YOLO.

Furthermore, **Figure 13** shows that the proposed DTL-YOLO model assigns higher confidence scores to correctly detected instances, indicating improved prediction reliability for damaged fresh tobacco leaves and further supporting the robustness of the proposed method.

**Figure 13:**
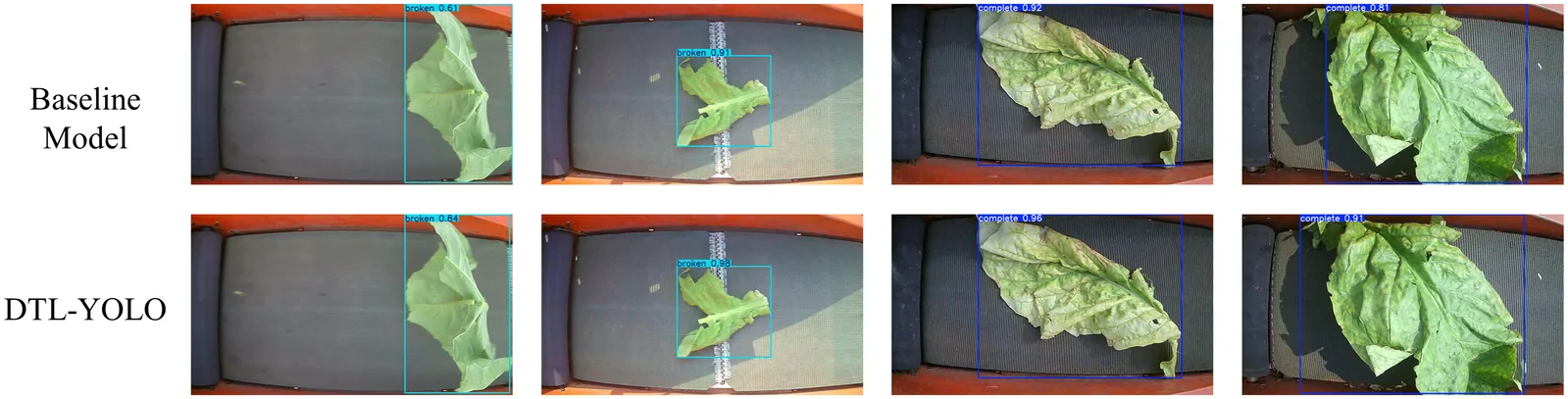
Representative prediction examples for intact and damaged leaves with confidence scores.

### 3.5 Deployment verification on embedded devices

To evaluate the on-device performance of the proposed model during mechanical tobacco harvesting, experiments were conducted on a Jetson Orin NX embedded platform under identical settings. For deployment, the trained DTL-YOLO model was exported from PyTorch to ONNX format and subsequently converted into a TensorRT engine for accelerated inference on the embedded platform. For all tests, the input resolution was fixed at 640×640 pixels. The optimized model achieved an inference speed of 24.7 FPS under the same input setting. As summarized in **Table 8**, DTL-YOLO reduced inference time from 51.02 ms to 40.49 ms and increased throughput from 19.6 to 24.7 FPS on the Jetson Orin NX. All evaluations were conducted on real video streams recorded during field operation of the layered tobacco harvester, and the deployment setup is shown in **Figure 14**.

**Table 8:** Performance comparison of DTL-YOLO and YOLOv8n on Jetson edge devices.

Model	Input size	Inference time/ms	FPS
YOLOv8n	640×640	51.02	19.6
DTL-YOLO	640×640	40.49	24.7

**Figure 14:**
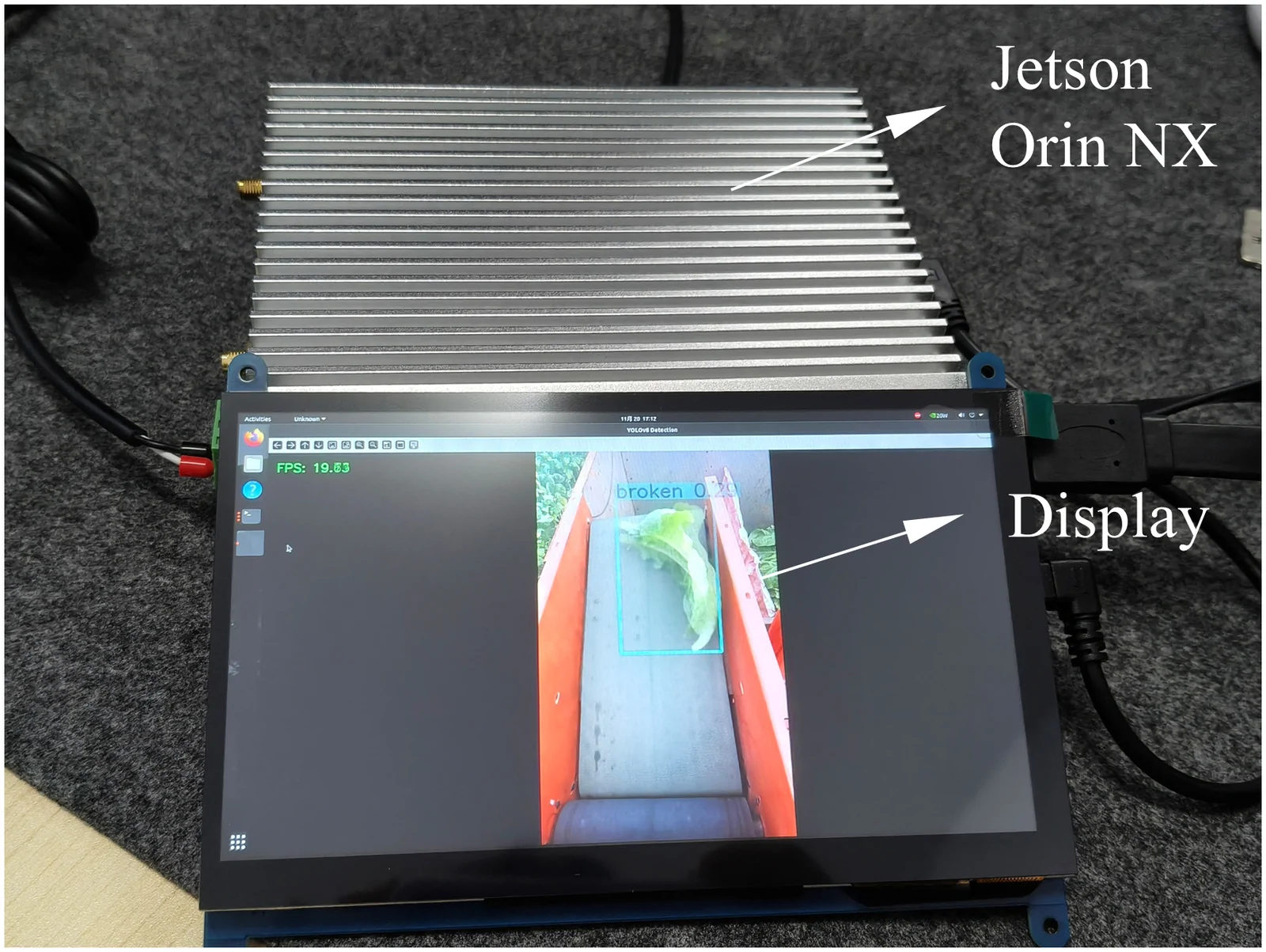
Experimental deployment of the detection models on the NVIDIA Jetson Orin NX platform.

The deployed system provides real-time detection results for intact and damaged tobacco leaves during field operation of the layered tobacco harvester. To obtain reliable leaf counts, a lightweight tracking-and-counting step was incorporated to maintain consistent leaf identities across consecutive frames and prevent duplicate counting; representative system outputs are provided in the Supplementary Video. Based on the counting results, the tobacco leaf damage rate can be calculated to provide information for subsequent harvesting quality assessment. These quantitative results may facilitate further evaluation of harvesting performance and support future optimization of harvesting operations. Experimental results show that, relative to the YOLOv8n baseline, the proposed model improves the on-device frame rate and reduces missed detections and false positives. These improvements indicate more reliable on-device damage detection, supporting the practicality of the optimized model on resource-constrained platforms and its capability to satisfy real-time performance demands under field harvesting conditions.

### 3.6 Discussion

The experimental results show that reducing model complexity while maintaining detection performance is an important consideration for fresh tobacco leaf damage detection. Replacing the original backbone with StarNet substantially reduces computational requirements but results in a modest decrease in detection performance. The subsequent incorporation of the improved BiFPN and ADown compensates for part of this loss by improving feature fusion and reducing information loss during downsampling. The four-level feature fusion combines detailed spatial information from shallow layers with contextual information from deeper layers, which facilitates the detection of damage regions with different sizes and visual characteristics. Therefore, the combined modifications provide a suitable balance between feature representation and computational efficiency for embedded deployment. The increased recall indicates that fewer damaged leaves were missed, which is particularly important for damage detection during harvesting. Meanwhile, the decrease in precision suggests a potential increase in false positive detections, reflecting the trade-off between detecting more potential damaged leaves and avoiding false alarms.

Nevertheless, field trials revealed several factors that may affect detection stability under practical harvesting conditions. Heavy leaf overlap and occlusion around the harvesting and conveying components can obscure damage characteristics and increase missed detections. Natural folds and local deformation may also resemble damaged regions and lead to false positive detections. In addition, tobacco leaves may bounce on the inclined conveyor belt under inertial forces during transportation. Combined with the relatively high conveying speed, unstable leaf motion may generate motion blur and reduce the number of valid frames captured within the camera field of view. Although visually interpretable frames containing motion blur were retained during dataset construction, severe blur and occlusion may still affect detection and subsequent tracking and counting. Therefore, improving detection stability under these complex harvesting conditions remains necessary for further field application.

Although the dataset was constructed using samples collected under both laboratory and actual harvesting conditions, broader field validation across different operating conditions remains necessary. Further evaluation across different cultivation regions, tobacco cultivars, harvesting periods, and harvester configurations would help to more comprehensively assess the generalization ability of the model. Future work will therefore expand the dataset using independent field samples and further investigate detection under severe occlusion and unstable leaf motion. In addition, independent harvesting trials combined with manually measured damage rates will be conducted to evaluate the influence of detection and counting errors on damage rate estimation and provide further validation for harvesting quality assessment.

## 4 Conclusions

In this study, a lightweight method for detecting mechanical damage in fresh tobacco leaves was developed by integrating StarNet, an improved BiFPN, and the ADown module into a YOLOv8n framework. On the self-collected dataset, the proposed model achieved 80.9% precision, 81.0% recall, and 88.4% mAP_50_. Compared with representative one-stage detectors (YOLOv5s, YOLOv8s, YOLOv9t, and YOLOv10n), DTL-YOLO provides a competitive balance between detection performance and model compactness. Relative to the YOLOv8n baseline, recall and mAP_50_ increased by 1.9 and 1.8 percentage points, while parameters, FLOPs, and model size decreased by 61.1%, 33.3%, and 58.7%. Deployment on a Jetson Orin NX platform further confirmed that the model can operate in real time under resource-constrained conditions, supporting its use for in-field monitoring during harvesting. Overall, within the scope of the YOLO-based detectors evaluated in this work, the proposed approach offers a practical solution for real-time damage detection in mechanically harvested fresh tobacco leaves and provides a visual basis for subsequent harvesting quality assessment. It also provides an engineering basis for future optimization of layered tobacco harvesters aimed at reducing mechanically induced damage.

## Data Availability

The raw data supporting the conclusions of this article will be made available by the authors, without undue reservation.
